# Time-Restricted Feeding and Aerobic Performance in Elite Runners: Ramadan Fasting as a Model

**DOI:** 10.3389/fnut.2021.718936

**Published:** 2021-09-21

**Authors:** Ali M. Al-Nawaiseh, Mo'ath F. Bataineh, Hashem A. Kilani, David M. Bellar, Lawrence W. Judge

**Affiliations:** ^1^College of Physical Education and Sport Sciences, The Hashemite University, Zarqa, Jordan; ^2^School of Sport Sciences, Jordan University, Amman, Jordan; ^3^Department of Applied Physiology Health & Clinical Sciences, University of North Carolina at Charlotte, Charlotte, NC, United States; ^4^School of Kinesiology, Ball State University, Muncie, IN, United States

**Keywords:** nutrition, running, intermittent fasting, time-restricted feeding, fasting (Ramadan)

## Abstract

A distance runner's performance is generally limited by energy availability when competing or training. Modifying meal frequency and timing by abstaining from eating or drinking, from dawn to dusk, during Ramadan fasting is hypothesized to induce hypohydration and reduced caloric and nutrient intake. The purpose of this study was to investigate the impact of Ramadan fasting on runners' performances. Fifteen trained male distance runners who observed Ramadan participated in this study (Age = 23.9 ± 3.1 years; Peak VO_2_ = 71.1 ± 3.4 ml/kg/min). Each participant reported to the human performance lab on two testing occasions (pre-Ramadan and the last week of Ramadan). In each visit, participants performed a graded exercise test on the treadmill (Conconi protocol) and their VO_2_, Heart Rate, time to exhaustion, RPE, and running speed were recorded. Detailed anthropometrics, food records, and exercise logs were kept for the entire period of the study. Repeated measure ANOVA, paired *t*-test, and Cohen's effect size analysis were carried out. Results indicated no significant influence for Ramadan fasting on body mass (*p* = 0.201), body fat (*p* = 0.488), lean body mass (*p* = 0.525), VO_2_max (*p* = 0.960), energy availability (*p* = 0.137), and protein intake (*p* = 0.124). However, carbohydrate (*p* = 0.026), lipid (*p* = 0.009), water (*p* < 0.001), and caloric intakes (*p* = 0.002) were significantly reduced during Ramadan Fasting. Daily training duration (*p* < 0.001) and exercise energy expenditure (*p* = 0.001) were also reduced after Ramadan. Time to exhaustion (*p* = 0.049), and maximal running speed (*p* = 0.048) were improved. Overall, time to exhaustion and maximal running speed of the distance runners was improved during Ramadan fasting, independent of changes in nutrients intake observed during the current study. With proper modulation of training, distance runners performance can be maintained or even slightly improved following the month of Ramadan fasting.

## Introduction

The popularity of endurance sports has increased globally in the last few decades, with the number of participants surpassing 5 million worldwide (1). A successful performance in such aerobic based events demands optimal nutritional regimens to allow for peak biochemical and physiological functions (2). Optimal physiological functions include energy production, transport of gases, removal of metabolic byproducts, and proper regulation of homeostasis. Competitive athletes under normal conditions strive to uphold proper nutrient intake, boost their energy storage, and maintain optimal hydration levels (3). Carbohydrate intake during exercise helps maintain high levels of carbohydrate oxidation, prevents hypoglycemia, and has a positive effect on the central nervous system.

Fasting for adults during the holy month of Ramadan is one of the mandatory practices in the Islamic religion; this annual event lasts for 30 consecutive days and mandates abstaining from eating or drinking from dawn to dusk. The number of daily meals is sometimes reduced, along with modified timing for such meals. Muslims taking part in Ramadan do not eat or drink anything during daylight hours, eating one meal (the “suhoor” or “sehri”) just before dawn and another (the “iftar”) after sunset. The modified meal timing is often referred to as intermittent fasting or time-restricted feeding (4–6). The restrictions on meal timing and frequency in conjunction with hypohydration during the daylight hours may impact Muslim athletes' ability to maintain proper nutrients and energy availability. Such conditions might be expected to add extra burden on athlete's ability to fulfill their nutritional requirements.

Meal consumption typically induces an increase in blood sugar level, which is regulated by insulin and glucagon. Recently, it has been reported that meal-consumption timing might influence energy balance (7, 8). Indeed, isocaloric meals with the same macronutrient content provided more calories when consumed at dinner when compared to breakfast. This might indicate that not only what we eat, but also when we eat, determines our physiological response to feeding and affects postprandial glucose levels.

Reduced energy and carbohydrate intake, impaired sleeping duration and timing, altered metabolic pattern, modified timing, and frequency of meal consumption, hypohydration, and oscillated circadian rhythm can all have unfavorable consequences on athletic performance (3). While some studies reported a detrimental effect of Ramadan fasting on athletes' performances, others showed minimal or no effects. However, in a recent study by Tinsley et al. (9) it is shown that, despite a reduction in caloric intake by 650 Kcal/day, the athletes revealed an increase in strength performance after 8 weeks of time restricted feeding (TRF). Furthermore, studies employing an intermittent fasting model for an extended period reported an enhanced neuromuscular performance in athletes (4, 9–11).

Studies that previously investigated the impact of Ramadan fasting on performance shared significant design and exercising protocol issues in common (12). Much of the available information on this topic has been collected from sedentary subjects or low-level competitors. Such design issues can be remedied by recruiting non-Muslim athletes and having them fast, or by recruiting Muslim athletes and having them only fast for a few days, which does not resemble fasting for the entire month of Ramadan. Additionally, to our knowledge, the impact of fasting on elite distance runners has not been investigated. The purpose of the current study is to investigate the effect of fasting during Ramadan (i.e., time-restricted feeding) on elite distance runners' performance. Moreover, the authors hypothesized that distance running performance will be positively impacted by Ramadan fasting in elite distance runners.

## Methods

### Experimental Approach

Fifteen trained male distance runners who observed Ramadan participated in this study. Each participant reported to the human performance lab on two occasions (pre-Ramadan and the last week of Ramadan). In each visit, participants performed a graded exercise test on the treadmill (13) and their VO_2_, Heart Rate, time to exhaustion, RPE, and running speed were recorded. Detailed anthropometrics, food records, sleep diary, and exercise logs were kept for the entire period of the study.

### Subjects

Fifteen trained male distance runners were recruited by word of mouth to participate in the present study (age = 23.9 ± 3.1 years; height = 170.9 ± 7.2 cm; body mass = 60.8 ± 6.4 kg; body mass index = 20.8 ± 1.3; Rest HR = 48.8 ± 7.9 bpm; VO_2Peak_ = 71.1 ± 3.4 ml/kg/min). Review of the similar published studies (13, 14) suggested that a sample size ≥ 12 would be sufficient for the present investigation. All participants were active runners (i.e., engaged in running activity for a minimum of 150 min/day, at least 5 days a week); however, none of the participants regularly performed resistance training exercise. After being informed of the procedures and potential risks involved in the investigation, an IRB-approved informed consent document was signed by each participant before the commencement of the study. Participants were not instructed to alter their training duration, intensity, or frequency. The daily fasting period was equal to or exceeding 15 h. The study was carried out during summer with an average maximum temperature during daylight hours of 34 C° and a mean relative humidity of 20% for the entire period of the study. During the study, subjects consumed a meal before dawn at ~0,500, with testing sessions beginning 4–4.5 h later.

### Procedures

The study was an observational design. All procedures were approved by the Hashemite University Institutional Review Board (IRB number 1520162017). Before the study, participants were informed of the study's purpose, along with any associated risks and benefits. In accordance with the university institutional review board, and the Declaration of Helsinki, participants gave written informed consent and completed a health history questionnaire before the first test session. All data was collected in the Human Performance Laboratory at Hashemite University. Participants completed three visits (a familiarization visit and two testing visits) on different days to undertake the study protocol as indicated below. On visit one (2 weeks before Ramadan), anthropometric characteristics, health status, blood pressure and heart rate were assessed. Briefly, participants were familiarized with the testing protocol and tools. Shortly after that, and following a 25–30 min resting period, the participants' blood pressure and resting heart rate (HR_rest_) measurements were conducted while seated, three readings were recorded, and their average was used to determine blood pressure and HR_rest_. On visit 2 (3 days before Ramadan: Pre-Fasting) and visit 33 (end of the fourth week of Ramadan: End-Fasting) preparations performed as described previously, and a Conconi (13) graded exercise test (GXT) to exhaustion began following a 15-min rest and a 5-to-7-min warm-up period. The initial speed was set at 8 km/h and increased by 1 km/h every 400 meters until exhaustion using a treadmill (Cosmos Saturn, Traunstein, Germany). All time to exhaustion running trials were performed at approximately the same time of day (between 10:00 am and 11:00 am), to avoid discrepancies in results caused by testing time. Oxygen consumption (VO_2_), and carbon dioxide production (VCO_2_) were calculated breath by breath using a respiratory metabolic cart (Quark PFT 2, COSMED. Rome, Italy). Heart rate and rate of perceived exertion (RPE) were obtained at the end of each stage (6–20, Borg scale chart). Participants' heart rate was monitored throughout the entire period of each trial using a chest strap heart rate monitor (Polar Electro, Oulu, Finland). Time to exhaustion was considered the point at which participants triggered the emergency stop, the termination signal, or failed to stay within safe range of the safety harness. Time to exhaustion was considered valid if participants achieved at least two of the following criteria at the point of exercise termination:

1. Plateau in VO_2_ with increasing speed.
2. Heart rate within 10 beats of the age predicted maximal heart rate.
3. RPE value more than or equal to 17.

### Body Composition

Body mass (BM) was measured with the use of a digital scale (SECA, Hamburg, Germany) to the nearest 0.1 kg wearing their running tops and shorts. Height was determined with the use of a stadiometer (SECA, Hamburg, Germany) to the nearest 0.1 cm. Measurements were compliant with the recommendations of the International Society for the Advancement of Kinanthropometry Guidelines. Body mass index (BMI) was calculated (kg/m^2^). Body fat (BF), body fat percentage, fat free mass (FFM), and total body water (TBW) were assessed with the use of four electrode bioelectrical impedance system (InBody720, Inbody Seoul, Korea). Reliability and validity of the measurement system has been reported in the literature (6). Anthropometric measurements were obtained on each visit after subjects rested for a period of 30 min following their scheduled arrival time and before beginning the warmup.

### Energy, Nutritional and Exercise Assessment

Participants were instructed to keep daily food records and exercise logs. The food records provided full details about ingested food and fluids along with time of consumption, preparation method, meal type, and perceived mood. Randomly selected complete food records were analyzed using NUTRITIONIST PRO (Axxya Systems; Woodinville, WA USA) and middle east food composition tables (http://www.fao.org/infoods/infoods/tables-and-databases/middle-east/en/). This software platform is routinely used in to validate other diet assessment tools (15). Exercise logs contained details about type of event, training duration, training intensity, and provided information about frequency of training. Exercise logs were analyzed using Sportlyzer (Sportlyzer LLC., Tartu, Estonia). Exercise energy expenditure (EEE) was estimated using the 2011 Compendium of Physical Activities (16). Energy availability (EA) was calculated as described by ACSM (17), and a good EA value was defined to be more than or equal to 45 kcal/kg FFM/day.

### Sleep Assessment

The *Daily* Sleep Diary (Loughborough University, Loughborough, UK) was used to evaluate each participant's subjective daily sleep score. The sleep diary consisted of a brief eight item questionnaire, evaluating the sleep time and quality. This and similar instruments have been reported on in the literature (18). During this study, sleep diary records were collected at two different times, one before (at the start of the study visit) and one during (at the end of study visit). The questionnaire was completed by each participant for the entire period of the study. The two sets of records were treated separately (i.e., not averaged) in order to assess reliability and to analyze fasting effect on sleep characteristics. Sleep diaries that show bedtime and awake time and daytime napping during the weekdays and at weekends was obtained together with the total sleep duration in a 24-h period (i.e., one full day) for the interval of the study. Comparison was administered for the difference in sleep duration before and during Ramadan.

### Statistical Analysis

All data is presented as means ± standard deviation (SD) and was analyzed using SPSS (version 25). Once the assumption of normality was confirmed using the Shapiro-Wilk test, parametric tests were performed. For anthropometric measures, sleep duration, and dietary variables, dependent sample *t*-test was used to detect significant differences between the two trials. For the RPE, VO2, and HR data, a two-way [Time (pre and end of Ramadan) x Speed] ANOVA with repeated measures was used. When appropriate, paired *t*-test was used for pair-wise comparisons. Cohen's *d* effect size for pairwise tests and partial eta squared (ηp^2^) for repeated measures analyses were calculated to assess the magnitude of difference between the time points. Effect sizes were considered small at 0.2, medium 0.5 and large at >0.8 for Cohen's *d* and 0.01 for small, 0.06 for medium and 0.14 for large for partial eta squared tests. Coefficient of variation were calculated for all measures and were all below 10% for performance measures. Dietary variables had higher variability (up to 53% for Protein intake), which was expected given the different size of the athletes and dietary preferences.

## Results

### Body Weight and Composition

Assessment of changes in body mass and body composition are presented in Table 1. There were no significant changes in body mass (*p* = 0.201), body fat (*p* = 0.488), and/or lean body mass (*p* = 0.525) due to Ramadan fasting.

**Table 1:** Assessment of changes in body weight and body composition during Ramadan fasting.

**Variable**	**Mean**	**SD**	***t***	***P*-value**	***d***
**Body weight (kg)**					
Pre-F	60.5	6.1	−1.347	0.201	0.36
End-F	60.8	6.4			
**Body fat (kg)**					
Pre-F	9.0	1.8	−0.714	0.488	0.19
End-F	9.1	1.9			
**Lean body mass (kg)**					
Pre-F	51.5	4.7	−0.653	0.525	0.17
End-F	51.7	5.1			

### Dietary Intake

Table 2 shows changes in nutrients intake while fasting during Ramadan. As expected, a significant decrease in carbohydrate intake, lipid intake, water intake, and caloric intake was detected at the end of Ramadan when compared with pre-Ramadan values with effect size ranging from medium to large (*d*: 0.67–1.46). However, protein intake was not significantly affected by Ramadan fasting.

**Table 2:** Assessment of changes in caloric, water, and macronutrients intake during Ramadan fasting.

**Variable**	**Mean**	**SD**	***t***	***P*-value**	***d***
**Carbohydrates intake gm/d**					
Pre-F	446.0	74.3	2.511	0.026	0.67
End-F	379.3	77.9			
**Proteins intake gm/d**					
Pre-F	118.1	54.4	1.643	0.124	0.44
End-F	88.6	53.3			
**Lipids intake gm/d**					
Pre-F	81.5	15.6	3.063	0.009	0.82
End-F	72.2	16.7			
**Energy intake Kcal/d**					
Pre-F	2,989.7	359.9	3.869	0.002	1.03
End-F	2,523.2	454.4			
**Daily water intake L/day**					
Pre-F	4.2	1.1	5.472	<0.001	1.46
End-F	2.5	0.8			

### Physical and Training Performance

A significant decrease was detected in daily training duration (*p* < 0.001; *d:* =2.98, 95% CI −16.4 to −40.8 min), and exercise energy expenditure (*p* = 0.001; *d:* = 1.18, 95% CI −198.0 to −609.9 Kcal) at the end of Ramadan compared with pre-Ramadan. Furthermore, time to exhaustion (pre: 1,392.9 ± 126.8 s vs. post: 1,448.4 ± 108.8 s, 95% CI 1.0–110.0 s) along with maximum speed (pre: 18.6 ± 2.3 km/h vs. post: 19.7 ± 1.9 km/h, 95% CI 0.0–2.27 km/h) were significantly improved toward the end of Ramadan (*p* = 0.04 and *p* = 0.048, respectively), with a medium effect size (*d:* 0.58–0.59). However, maximum oxygen consumption, and energy availability were not significantly affected by Ramadan fasting (*p* = 0.960 and *p* = 0.137 respectively).

### RPE, Oxygen Consumption, and Heart Rate Before and During Ramadan Fasting

**Figure 1:**
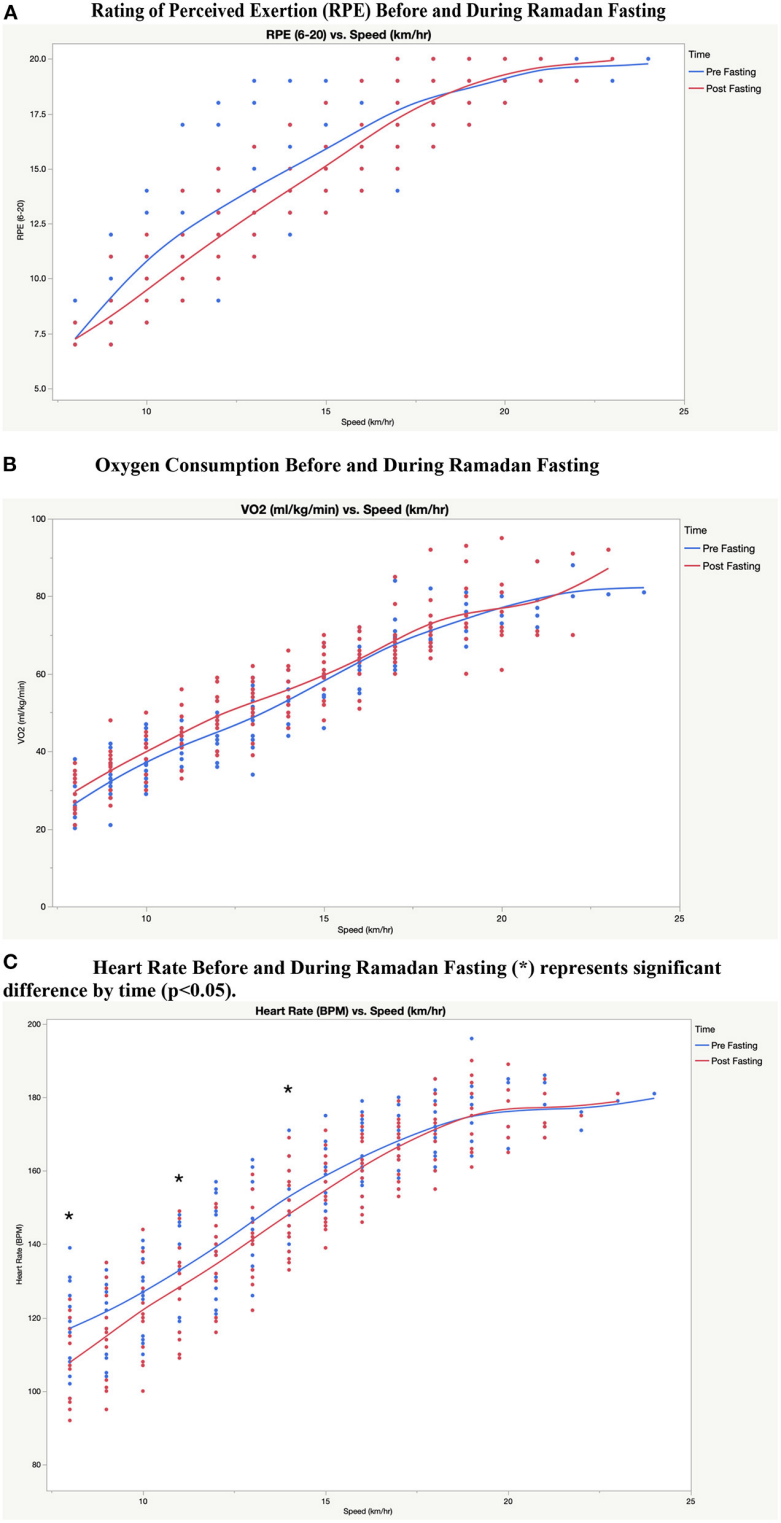
**(A)** Rating of Perceived Exertion (RPE) before and during Ramadan fasting. **(B)** Oxygen Consumption Before and During Ramadan Fasting. **(C)** Heart Rate Before and During Ramadan Fasting (*) represents significant difference by time (*p* < 0.05).

Figures 1A–C shows the changes in RPE, Oxygen consumption, and heart rate, at different running speeds before and during Ramadan fasting. The repeated-measures analysis of variance indicated no significant main effects for time on VO2 values at all running speeds (*p* > 0.05). Additionally, no significant effect was observed for time on RPE (*p* > 0.05) at all running speeds. Repeated-measures ANOVA revealed a significant main effect for time [*F*_(1,4)_ = 10.689, *p* = 0.031, ηp^2^ = 0.728] on heart rate values. Heart rate values were significantly lower at low and medium-high speeds (8 km/h, *p* = 0.001 pre: 117.3 ± 12.0 vs. post: 107.9 ± 10.9; 11 km/h, *p* = 0.016 pre: 133.0 ± 10.8 vs. post: 127.9 ± 13.5; 14 km, *p* = 0.030 pre: 153.6 ± 10.4 vs. post: 148.5 ± 11.5) during Ramadan as compared to pre-Ramadan, however, the values were comparable at higher speeds.

### Sleep

**Figure 2:**
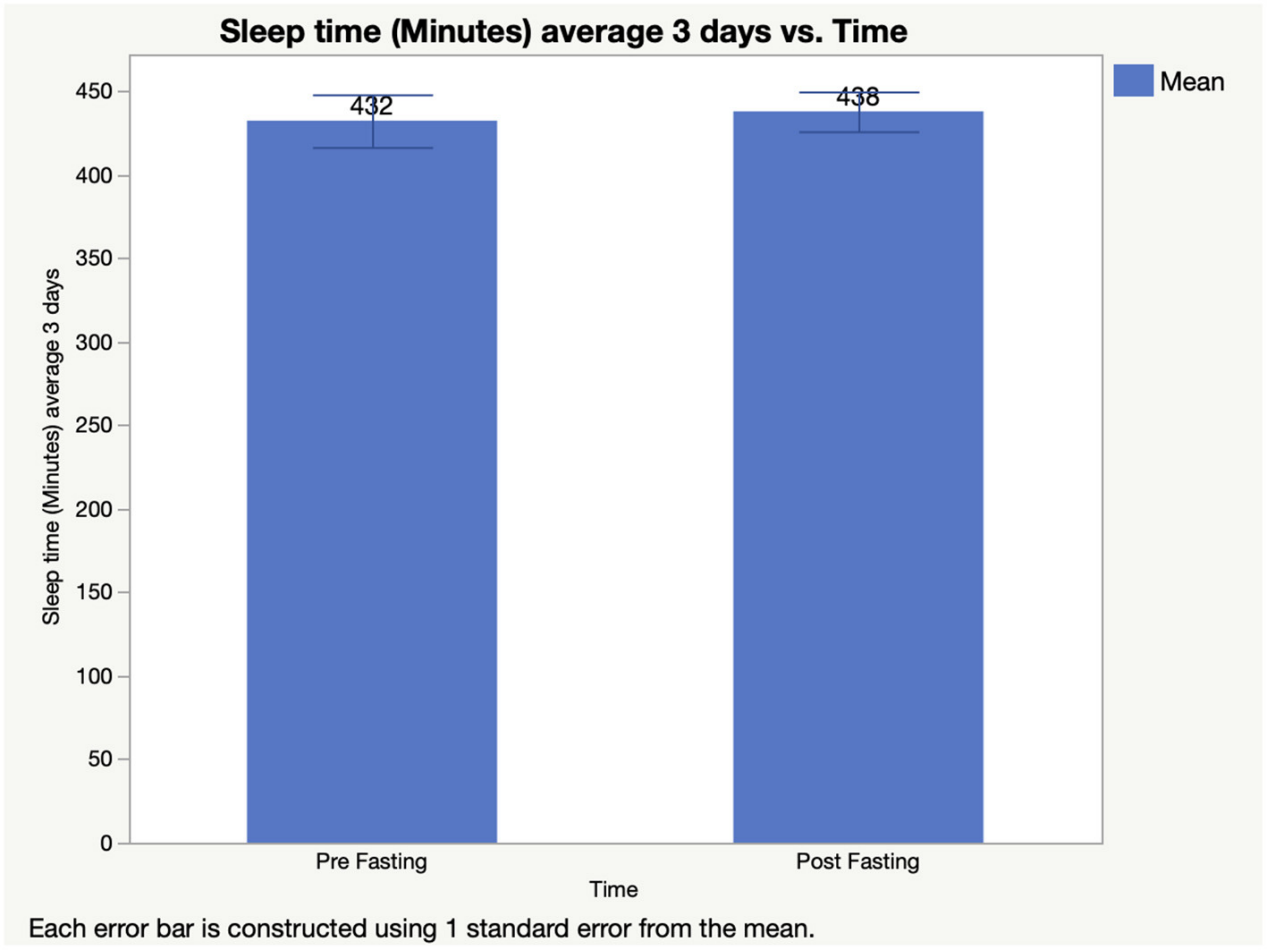
Sleeping Duration Before and During Ramadan Fasting.

Figure 2 reports changes in sleep time because of Ramadan fasting. Sleep durations were comparable during Ramadan (437.6 ± 44.5 min/24 h) in comparison with before Ramadan (432.0 ± 59.0 min/24 h) (*t* = −0.363; *p* = 0.722; *d* = 0.108, 95% CI −27.5–38.7 min).

## Discussion

This study investigated the impact of Ramadan-Fasting on elite distance runners' performance. The results showed that time to exhaustion and running speed were improved under fasting conditions in comparison to non-fasting. However, the performance improvements observed among fasting athletes in the current study were independent of changes in Peak VO_2_, oxygen consumption, or RPE. Furthermore, energy and water intake were significantly reduced during Ramadan. Macronutrients intake (except for protein) were also reduced during Ramadan.

In previous studies, Chtourou (14, 19) observed an attenuated physical performance in long-distance running during Ramadan fasting. A possible explanation for such a conflicting outcome with current results may be attributed to the employed testing protocol. Indeed, studies that employed a graded exercise test (20, 21), similar to current design, reported a better outcome in comparison to protocols comprised of mere constant intensity. Additional explanation for this disparity in results might also be attributed to athletic level and discipline (4, 21, 22). Elite athletes tend to react to the conditions of Ramadan fasting in a different manner compared with other categories of athletes, suggesting that a higher level of adaptation may protect against performance decay during extended periods of unfavorable conditions (23–27).

A significant improvement in time to exhaustion at the end of Ramadan fasting compared to pre-Ramadan was detected in this study. A similar result was observed in a study that was carried out during Ramadan fasting with Turkish soccer players (20), which showed an increase in total running time in the last week of Ramadan during the modified 20 m shuttle running test (MSRT) performed to volitional fatigue. Furthermore, the current study showed a pronounced reduction in daily training duration (~48% compared with Pre-Ramadan) while maintaining the number of weekly training sessions. Thus, the improved time to exhaustion might be attributed to a form of maintenance-like training. In support, Garci (28) concluded that 2 weekly endurance training sessions would be enough to maintain aerobic power under conditions of reduced training.

The 6% improvement in maximum running speed observed in the current study was in agreement with previous findings reported by Güvenç (20). The researcher studied the effect of Ramadan fasting on aerobic performance in a sample of athletes comparable in age and fitness level to current study participants. The study reported an increase in peak running velocity toward the end of the fasting month compared with pre-Ramadan. The same author also reported a decrease in peak running velocity during the first week of Ramadan fasting compared with pre-Ramadan. These findings might suggest that athletes tend to positively adapt to Ramadan fasting toward the end of the month. Indeed, several published studies that investigated the effect of Ramadan fasting reported a negative influence on endurance performance when exercise testing was carried out within the first two weeks of Ramadan fasting (22, 29).

The current study showed a lack of a significant change in Peak VO_2_ despite a significant reduction in training volume during the fasting period. The current results are consistent with the results of a previous study (28) that reported consistency in VO_2_ max in world class non-fasting Kayakers following 5 weeks of reduced training, preceded by a period of tapering for 4 weeks. Such findings might suggest that a reduction in training volume for the entire period of the holy month of Ramadan should not alter maximal aerobic power from a scientific and training standpoint.

The current study results showed comparable values for oxygen consumption along with lower heart rate values when comparing pre-Ramadan with Ramadan fasting values while performing the graded exercise test. These findings agree with previously published results that also show no significant changes in oxygen consumption and lower heart rate because of Ramadan fasting (20, 30). In contrast, short-term fasting (i.e., overnight fasting) did not alter heart rate during the 6-min walk test in athletes in comparison with a non-fasting state (1, 31). The findings in Ramadan fasting athletes may suggest that prolonged fasting periods have different effects on aerobic performance indicators in comparison with either shorter fasting periods or non-fasting athletes. Similarly, RPE values in the current study were not altered across all running speeds due to Ramadan fasting, which was in contrast with previous studies. The disparity in RPE values between the current study and previous studies may be attributed to the fact that participants' fasting in the current study was voluntarily conducted, thus it may not have added an extra psychological burden. The lack of an increase in RPE during Ramadan fasting may also explain the improved time-to-exhaustion values achieved by fasting participants in the current study. This ability to outperform non-fasting values can be justified by psychological means such as Hormesis (32–34).

In the present study, body mass, body fat mass, and lean body mass, despite the long hours of Ramadan fasting, were not significantly changed (Table 1). Concerning dietary pattern and energy expenditure, the present study showed a significant reduction in carbohydrates, lipids, water, and caloric intake. The absence of changes in body mass and composition can be explained by our findings which identified a decline in daily training duration and intensity and the reduced exercise caloric expenditure which resulted in a comparable energy availability value. This result was in agreement with results from previous studies that reported no change in body mass during Ramadan fasting when comparable energy availability values were maintained (31, 35). Additionally, Mujika (36) suggested that athletes and trained subjects could maintain their lean body mass and muscular strength after reducing training loads. They also projected that the reduced training load could decrease the risk of dehydration (i.e., reduction in the sweat losses during training sessions). The reduced risk of dehydration may help athletes to preserve their energy levels for quality workouts and training (36).

As shown in Figure 2, sleeping duration was not changed between pre-Ramadan and during Ramadan for our group of athletes. This represents a difference between athletes and non-athletes who observed Ramadan from a similar age group that demonstrated reduced total sleeping hours during Ramadan (37). Muslim athletes managed to maintain their regular sleep duration, based on the results of the present study.

It is important to acknowledge the strengths and limitations of the current research. To date, the impact of fasting on elite distance runners has not been investigated. Limitations for the present work include sample size, lack of a control group, lack of data on training intensity and reliance on some self-reported data. A larger sample would have served to increase the statistical power for this research. A control group would have allowed for the effect of training to be determined on the study outcomes, however; given the mandatory nature of Ramadan fasting a control group of similar individuals was not feasible. The success of food records and exercise logs depends on the memory, cooperation, and the communication ability of the subject(s). Participants may have attempted to present themselves and their answers in a more positive light, and participants potentially may have worried about the perceived repercussions caused from their responses.

## Conclusions

It is important that individual sport coaches are accommodating toward various cultural and/or religious values and are mindful of the potential impact certain dietary practices may have on an athlete. Fasting during the holy month of Ramadan is one of the mandatory practices in the Islamic religion. The restrictions on meal timing and frequency in conjunction with hypohydration during the daylight hours pose a challenge for the Muslim athlete to be able to maintain proper nutrients and energy availability for training. Given the results of the present study, a strategy of adjusting training intensity and duration may be considered to help maintain the quality of training during times of fasting. The present study also suggests that training might be optimized if it occurs during the morning hours approximately 4 h after the pre-dawn meal. The results of the present study showed a lack of a significant change in Peak VO_2_, despite a significant reduction in training volume, and time-to-exhaustion and running speed were actually improved under fasting conditions. Despite the long hours of Ramadan fasting, body mass, body fat mass, and lean body mass, were not significantly changed. Therefore, a decline in daily training duration and intensity and the resultant reduced exercise caloric expenditure may explain the promising results in the present study. Coaches must closely monitor training results and make adjustments as needed to ensure their athletes are being properly prepared for training and competition in the most effective and safe manner.

## Data Availability Statement

The raw data supporting the conclusions of this article will be made available by the authors, without undue reservation.

## Ethics Statement

The studies involving human participants were reviewed and approved by HASHEMITE UNIVERSITY. The patients/participants provided their written informed consent to participate in this study.

## Author Contributions

AA-N: conception and design of study and data acquisition. MB: data collection and design of study. HK: data interpretation and critical revisions. DB: statistical analysis and data interpretation. LJ: critical revisions and data interpretation. All authors contributed to the article and approved the submitted version.

## Conflict of Interest

The authors declare that the research was conducted in the absence of any commercial or financial relationships that could be construed as a potential conflict of interest.

## Publisher's Note

All claims expressed in this article are solely those of the authors and do not necessarily represent those of their affiliated organizations, or those of the publisher, the editors and the reviewers. Any product that may be evaluated in this article, or claim that may be made by its manufacturer, is not guaranteed or endorsed by the publisher.
